# Sex stratified analysis of patients with resistant hypertension from the Global SYMPLICITY Registry of renal denervation

**DOI:** 10.1038/s41440-025-02446-y

**Published:** 2025-11-20

**Authors:** Anastasia S. Mihailidou, Felix Mahfoud, Markus Schlaich, Roland Schmieder, Krzysztof Narkiewicz, Luis Ruilope, Martin Fahy, Michael Böhm, Giuseppe Mancia, Laura Nickel, Douglas A. Hettrick, Joachim Weil

**Affiliations:** 1 Cardiology Department, Royal North Shore Hospital, Sydney, NSW Australia; 2 Cardiovascular & Hormonal Research, Kolling Institute, Sydney, NSW Australia; 3 Department of Cardiology, University Heart Center, University Hospital Basel, Basel, Switzerland; 4 Cardiovascular Research Institute Basel (CRIB), University Heart Center, University Hospital Basel, Basel, Switzerland; 5 Dobney Hypertension Centre, Medical School –Royal Perth Hospital Unit, Perth, WA Australia; 6 University Hospital Erlangen, Erlangen, Germany; 7 Medical University of Gdansk, Gdansk, Poland; 8 Hospital Universitario 12 de Octubre and CIBERCV and School of Doctoral Studies and Research, Universidad Europea de Madrid, Madrid, Spain; 9 Medtronic PLC, Santa Rosa, CA USA; 10 Saarland University Hospital, Homburg/Saar, Germany; 11 University of Milano-Bicocca, Milan, Italy; 12 Sana Kliniken Lübeck GmbH, Lübeck, Germany

**Keywords:** Blood pressure, Women, Phenotype, Implemental hypertension

## Abstract

Our aim was a sex-specific analysis to characterize the phenotype for women with resistant hypertension (rHTN), an understudied population, referred for renal denervation (RDN) from the Global SYMPLICITY Registry DEFINE (*N* = 3332 patients). For this analysis, 2502 patients with uncontrolled hypertension (office systolic blood pressure (SBP) ≥140 mmHg, ≥3 antihypertensive drugs) were referred for RDN. Age at baseline was 18–88 years for men and 21–89 years for women. We used propensity score matching to account for demographic differences at baseline identified by multivariate regression analysis. Changes in BP, outcomes (all-cause death, cardiac death, stroke, myocardial infarction), and quality of life (QoL) after 36 months were assessed. Women had fewer comorbidities at baseline but had higher BP and worse QoL, anxiety, and depression compared to men. After propensity matching to minimize bias, BP changes were comparable by sex, and BP was significantly reduced from baseline following RDN. Women ≥55 years of age with resistant hypertension had a greater reduction in BP compared with women <55 years. At 36 months after RDN, there was a significant 12% reduction in anxiety/depression compared to baseline for women with resistant hypertension. When baseline office SBP was in the highest tertile (>178 mmHg), cardiac death was more prevalent in women (6.1%) than men (1.7%). Our sex-stratified analysis of the global registry allowed a longitudinal assessment, providing important insights into the phenotype of resistant hypertension in women. We identified sex-specific differences that highlight the need for early detection and management of hypertension in women.

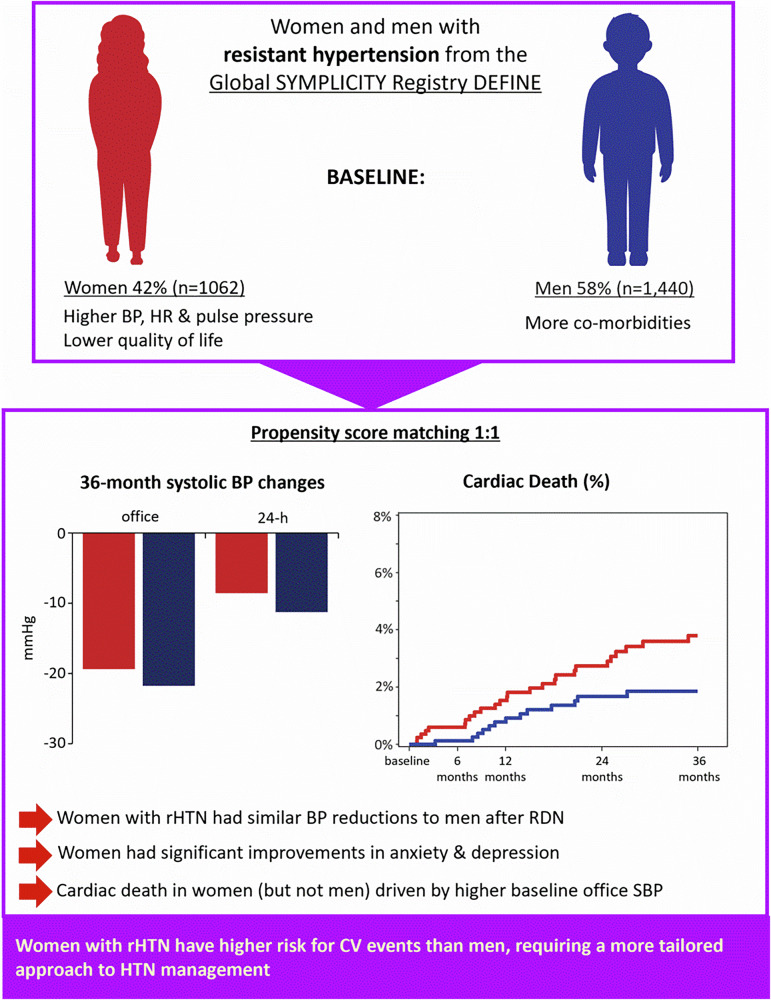

## Introduction

Hypertension is the major risk factor for cardiovascular disease (CVD) mortality for women globally [1]. For every 10 mmHg increase in systolic blood pressure (BP), the risk of CVD is higher for women (25%) than men (15%) [2]. Midlife hypertension is more common in men than women; however, women have an increased hypertension-associated risk of dementia starting early in the 4th decade [3]. Several studies show CVD risk starts at lower levels of blood pressure for women than men, including risk of myocardial infarction, stroke, and heart failure [4–6]. Whereas age-standardized prevalence of having 5 or more comorbidities has been shown to be higher for women than men [7].

Women have a greater risk of CVD mortality with high SBP than men [8]. There are also sex differences in the pharmacokinetics of antihypertensive medications, with women having higher exposure and more frequent adverse drug reactions [9] and side effects resulting in hospitalization [10]. Sex differences in adherence have also been reported with either no association [11] or women having lower adherence than men [12, 13]. With regard to medication persistence, women have higher rates; however, this changes with severe hypertension, and men have higher persistence [14]. The aim of this analysis was to evaluate outcomes in patients with resistant hypertension (rHTN) after RDN based on sex, which to date has not been well characterized. We used a large global registry of real-world patients with hypertension referred for RDN.

## Methods

### Study design

The Global SYMPLICITY Registry DEFINE study design has been previously described (www.clinicaltrials.gov: NCT01534299) [15, 16]. It is a prospective, multi-center, all-comer, single-arm, open-label, observational, global study designed to evaluate the safety and efficacy of radiofrequency RDN in a real-world, clinical setting. Patients with uncontrolled hypertension were enrolled, and all patients provided written informed consent. Baseline demographics were recorded in electronic case report forms (eCRF), including eGFR (calculated using the MDRD formula). QoL was assessed using the EQ-5D-3L questionnaire and the methods previously reported [17]. This study adheres to the Declaration of Helsinki and was approved by each enrolling center’s institutional review board or ethics committee.

### Study procedures

Radiofrequency RDN was performed on all patients using the previous generation, single-electrode Symplicity Flex^TM^ or the current generation, 4-electrode Symplicity Spyral^TM^ catheter (Medtronic plc, Santa Rosa, CA, USA). Details of the procedure have been described previously [18, 19]. Patients were followed at 6-, 12-, 24-, and 36-month post-procedure per standard of care. Office and 24-h ambulatory BP were measured at baseline, discharge, and at each follow-up according to the Seventh Report of the Joint National Committee on Prevention, Detection, Evaluation, and Treatment of High Blood Pressure guidelines [20]. BP changes were also evaluated in women by age groups approximating pre- and postmenopausal: <55 and ≥55 years old. Prescribed antihypertensive medication classes were recorded for each follow-up. Clinical outcomes, including myocardial infarction (MI), stroke, cardiovascular death, and all-cause death, were recorded through 36 months. Adverse events were adjudicated independently by the Clinical Events Committee (Cardiovascular Research Foundation, New York, NY, USA).

### Statistical analysis

We analyzed patients from the GSR DEFINE with rHTN defined as office SBP ≥ 140 mmHg and prescribed ≥3 antihypertensive drugs at baseline. Continuous variables are reported as mean ± standard deviation (SD), and categorial variables are reported as percentages. SAS for Windows 9.4 (SAS Institute, Cary, NC, USA) was used for all statistical analyses. The propensity matched analysis used the Greedy-Match algorithm and used baseline demographic variables that were significantly different by sex and/or considered important variables that influenced BP. This included age, baseline office SBP, history of sleep apnea, cardiac disease, smoking history, and baseline number of antihypertensive drugs.

Comparisons in baseline demographics and clinical outcome rates by sex used a two-sample t-test or a Pearson chi-squared test, depending on the distribution. Comparisons of BP reductions were by analysis of covariance (ANCOVA) adjusted for baseline BP. Comparison of QoL between baseline and 36-month measures was analyzed by McNemar’s test. Propensity-matched groups were evaluated for BP changes and clinical outcomes comparing women and men. A multivariate stepwise selection algorithm was performed to identify significant covariates that correlated with BP changes at 36 months for women with rHTN. This identified significant covariates from the following with an entry/inclusion criteria *p* ≤ 0.10: age, baseline SBP, number of antihypertensive drugs, body mass index, cardiac disease, diabetes, baseline heart rate, sleep apnea, hypertension, atrial fibrillation, history of combined hypertension, smoking history, and baseline eGFR. Outcomes by baseline office SBP tertiles in women were also evaluated (Lower BP tertile: <160 mmHg, middle BP tertile: 160 to 178 mmHg, upper BP tertile: office SBP > 178 mmHg). We also performed an additional sub-analysis for patients with refractory hypertension, defined as baseline office SBP ≥ 140 mmHg and prescribed ≥5 antihypertensive medications, including a mineralocorticoid antagonist. Although very few patients referred for RDN in the GSR had diastolic rHTN with controlled SBP, we performed a post hoc analysis of patients from the full GSR cohort with isolated diastolic hypertension defined as baseline office SBP < 140 mmHg and DBP ≥ 90 mmHg.

## Results

### Baseline demographics and blood pressure

As of March 2023, there were 2502 patients (42% [1062] women and 58% [1440] men) with rHTN, enrolled in GSR DEFINE. Baseline characteristics of rHTN patients, including comparisons by sex, are provided in Table 1. Women were older than men (62 ± 13 vs. 59 ± 12 years; *p* < 0.0001) and had fewer baseline comorbidities, including sleep apnea (12.5% vs. 25.0%; *p* < 0.0001), cardiac disease (45% vs. 50%; *p* = 0.012), and smoking history (19.1% vs. 41.3%; *p* < 0.0001). While women had slightly lower eGFR than men (74.3 ± 25.6 vs 77.1 ± 25.2 mL/min/1.73 m²; *p* = 0.0005), there were no differences in chronic kidney disease or patients with eGFR <60 ml/min/1.73 m^2^. The history of renal stenosis was not systematically assessed and was based on the reporting in the eCRF. Patients with significant renal artery stenosis detected during or immediately prior to the procedure >50% are considered ineligible or RDN.

**Table 1:** Baseline demographics of patients

	Total (*n* = 2502)	Male (*n* = 1440)	Female (*n* = 1062)	*P*-value
Age	60.2 ± 12.1	59.2 ± 11.6	61.7 ± 12.7	<0.0001
BMI (kg/m2)	31.1 ± 5.7	31.1 ± 5.2	31.0 ± 6.3	0.48
History of kidney disease (%)	29.9	29.5	30.5	0.57
Renal insufficiency (eGFR < 60 mL/min/1.73 m²)	20.7	20.1	21.4	0.44
eGFR (mL/min/1.73 m²)	75.9 ± 25.4	77.1 ± 25.2	74.3 ± 25.6	0.0005
Microalbuminuria/proteinuria (%)	7.1	8.0	6.0	0.051
Known renal artery stenosis (%)	2.7	2.1	3.6	0.022
Hemodialysis (%)	0.9	1.3	0.4	0.021
Sleep apnea (%)	19.7	25.0	12.5	<0.0001
Vascular disease (%)	24.9	24.5	25.5	0.58
Cardiac disease (%)	48.1	50.3	45.2	0.011
Atrial fibrillation (%)	12.1	11.9	12.5	0.64
Diabetes (%)	42.9	44.0	41.4	0.20
Type 1	2.8	3.3	2.2	0.086
Type 2	39.4	40.6	39.2	0.49
Hypercholesterolemia (%)	38.3	37.4	39.6	0.25
Smoking history (%)	31.9	41.3	19.1	<0.0001
Pulse pressure (mmHg)	78.9 ± 19.5	76.3 ± 18.2	82.4 ± 20.7	<0.0001
Office heart rate (bpm)	70.6 ± 13.2	69.4 ± 13.0	72.1 ± 13.2	<0.0001
Office systolic BP (mmHg)	171.2 ± 21.4	168.9 ± 19.4	174.2 ± 23.4	<0.0001
Office diastolic BP (mmHg)	92.3 ± 16.3	92.6 ± 15.4	91.8 ± 17.4	0.096
24-h ambulatory heart rate (bpm)	68.7 ± 11.4	67.4 ± 10.9	70.4 ± 11.9	<0.0001
24-h ambulatory systolic BP (mmHg)	156.1 ± 18.5	155.2 ± 17.2	157.4 ± 20.2	0.035
Daytime systolic BP (mmHg)	158.7 ± 18.4	157.8 ± 17.3	159.9 ± 19.9	0.047
Nighttime systolic BP (mmHg)	148.1 ± 21.4	147.4 ± 20.3	149.2 ± 22.8	0.080
24-h ambulatory diastolic BP (mmHg)	87.7 ± 14.6	89.0 ± 13.8	85.8 ± 15.5	<0.0001
Daytime diastolic BP (mmHg)	90.0 ± 15.0	91.2 ± 14.2	88.3 ± 16.0	<0.0001
Nighttime diastolic BP (mmHg)	80.8 ± 15.0	82.4 ± 14.4	78.5 ± 15.5	<0.0001

Women with rHTN had higher baseline office SBP (174 ± 23 vs 169 ± 19 mmHg, *p* < 0.0001) and 24-h ambulatory SBP (157 ± 20 vs. 155 ± 17 mmHg; p = 0.035) compared with men (Table 1). On the other hand, 24-h ambulatory diastolic BP (DBP) but not office DBP, was lower in women than men (86 ± 16 vs. 89 ± 14 mmHg, respectively; *p* < 0.0001). Pulse pressure in women was higher than in men (82 ± 21 vs 76 ± 18 mmHg; *p* < 0.0001). Women had a higher heart rate, both office (72 ± 13 vs. 69 ± 13 bpm; *p* < 0.0001) and during 24-h ambulatory monitoring (70 ± 12 vs. 67 ± 11 bpm; *p* < 0.0001), compared with men. For the RDN procedure, there were no differences in procedure time, access site, denervation time, number of branch vessels treated per patient, and contrast volume. Due to numerous baseline characteristics that differed between women and men, we performed a propensity-matched analysis to compare outcomes between sexes.

### Propensity-matched analysis

**Fig. 1:**
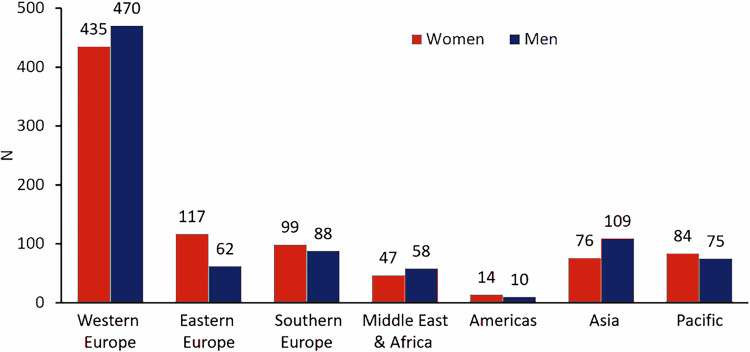
Enrollment of men and women in the Global SYMPLICITY Registry DEFINE by geographical regions in propensity score-matched groups. Western Europe: Austria, Switzerland, Belgium, Netherlands, Denmark, Sweden, France, Germany, UK, Ireland; Eastern Europe: Czech Rep, Hungary, Latvia, Lithuania, Poland, Slovakia, Ukraine, Kazakhstan, Belarus, Russia; Southern Europe: Italy, Spain, Portugal, Greece; Middle East & Africa: Kuwait, UAE, South Africa, Tunisia, Israel, Saudia Arabia, Egypt; Americas: Canada, Mexico, Colombia, Brazil, Argentina; Asia: China, Hong Kong, Taiwan, Malaysia, Thailand, Singapore, South Korea and Bangladesh; Pacific: Australia, New Zealand

The propensity-matched cohort included 1744 patients (872 female: 872 male) matched to the baseline demographic variables that were significantly different and/or considered important variables influencing BP. The number of patients for each individually matched variable is shown in Supplementary Table S1. Recruitment rates for this registry are shown in Fig. 1, with each region enrolling a similar proportion of male: female patients and truly a global representation, with Western Europe having the highest recruitment rate. A similar proportion of patients were prescribed different classes of antihypertensive drugs in the propensity-matched cohorts at baseline. At 36 months post RDN, the number of antihypertensive drug classes was comparable by sex (female 4.7 ± 1.6 vs male 4.7 ± 1.6), although fewer women with rHTN were prescribed calcium channel blockers than men (76% vs 82% respectively; p = 0.026; Supplementary Table S2**)**.

### Blood pressure changes in propensity-matched cohorts

**Fig. 2:**
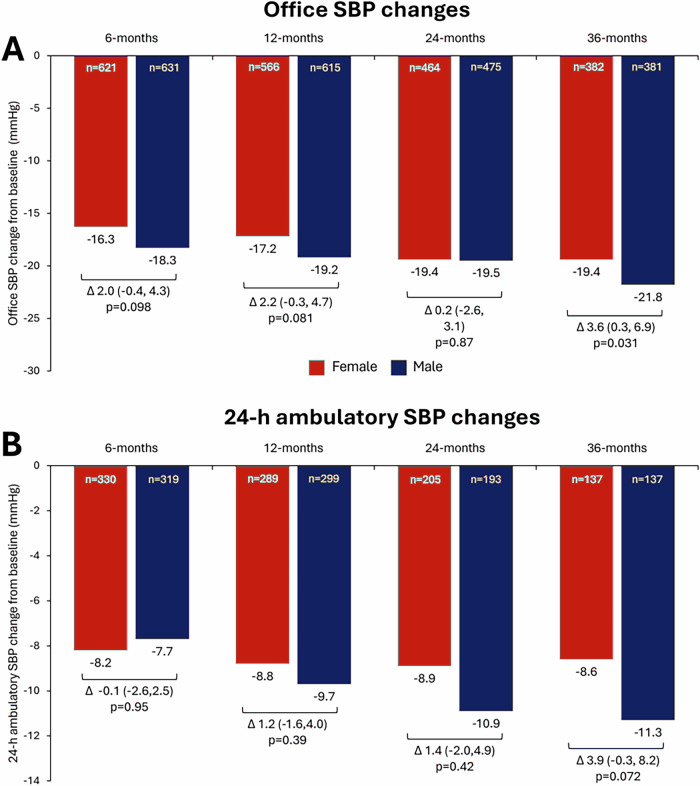
Office and 24 hr SBP changes through 36 months in the propensity-matched cohort with resistant hypertension by sex. **A** office SBP change, **B** 24-h SBP change. All SBP changes from baseline are *p* < 0.0001. The *p*-values above comparing SBP changes by sex are ANCOVA adjusted for baseline systolic BP. SBP Systolic blood pressure

There were progressive reductions in office and 24-h ambulatory SBP through to 36 months after RDN in both sexes after propensity matching (Fig. 2). Reductions in office SBP were similar among men and women through 24 months. At 36 months, there was an ANCOVA-adjusted difference of 3.6 mmHg in office SBP between women and men (female -19 ± 30 vs male -22 ± 24 mmHg; p = 0.031; Fig. 2A). The difference in 24-h ambulatory systolic BP changes at 36 months was 3.9 mmHg (female −9 ± 22 vs male −11 ± 20 mmHg; *p* = 0.072; Fig. 2B**)**. At 36 months, there was no longer a significant difference in systolic dipping patterns by sex. Similarly, there were significant reductions in office and 24-h ambulatory DBP in both sexes through to 36 months (Supplementary Fig. S1).

Sub-analysis of 479 patients in this propensity-matched cohort, which met the definition of refractory hypertension at baseline (19% of the full cohort), showed similar SBP and DBP between men and women at baseline and at 3 years (Supplementary Table S4). The drop in BP from baseline was significant at 3 years in both groups and was not different between groups. Post-hoc analysis found only 39 patients (1%) met the definition of isolated diastolic hypertension (Supplementary Table S5). Among patients with available 3-year data (*N* = 18), there was no difference in pressures between males and females.

In these propensity-matched groups, baseline nighttime SBP was similar (female 148 ± 22 vs male 146 ± 20 mmHg; p = 0.13). Twenty-four hour circadian BP dipping patterns (i.e., the ratio of nighttime BP relative to daytime BP) were different between sexes. At baseline, a smaller proportion of women compared with men were non-dippers (e.g., men had relatively higher nighttime BP; defined as night-day BP ratio >0.9 and ≤1.0) in their circadian BP pattern (female 41% vs male 50%; *p* = 0.0034).

We also investigated BP changes in women by age. At 36 months after RDN, women with rHTN in the ≥55 years old age group, compared with women in the <55 years old age group, had greater office SBP reduction (−21 ± 29 vs -15 ± 32 mmHg; adjusted *p* = 0.0013; Supplementary Fig. S2A), as well as 24-h ambulatory SBP reduction (−11 ± 20 vs -3 ± 26 mmHg; adjusted *p* = 0.0031; Supplementary Fig. S2B).

### Baseline characteristics associated with BP changes

A multivariate stepwise selection algorithm showed that at 36 months, baseline office SBP and age were independently associated with office SBP changes among women. Specifically, for every 1 mmHg increase in baseline office SBP, there was a decrease of 0.8 mmHg (*p* < 0.001), and for every 1-year increase in age, there was a decrease of 0.2 mmHg (*p* = 0.044). In addition, only baseline 24-h ambulatory SBP in women was independently associated with 24-h ambulatory SBP changes. For every 1 mmHg increase in baseline 24-hour ambulatory SBP, there was a decrease of 0.2 mmHg (*p* = 0.006; Supplementary Table 3).

### Outcomes

**Fig. 3:**
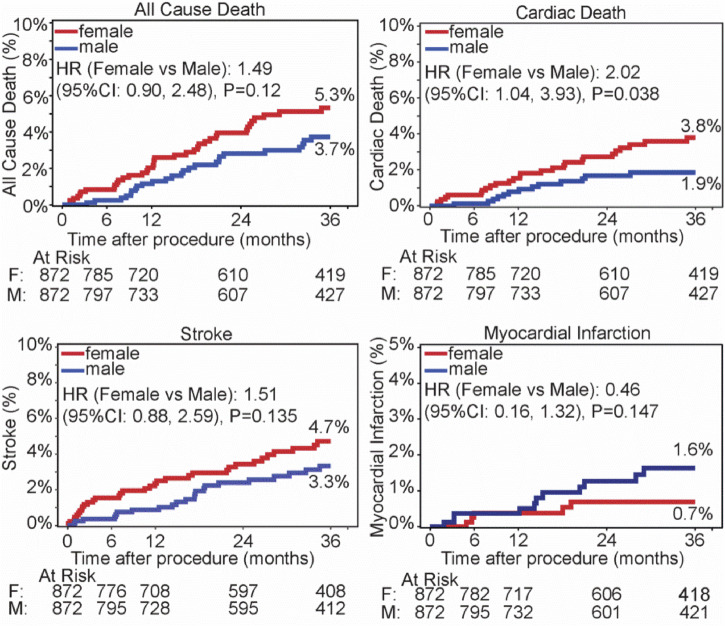
Outcomes through to 36 months in resistant hypertension patients. Women and men were propensity-matched. Kaplan Meier survival estimates were compared by sex for the rates of: all cause death (upper left), cardiac death (upper right), stroke (lower left), myocardial infarction (lower right). Cardiac death includes an unknown death

**Fig. 4:**
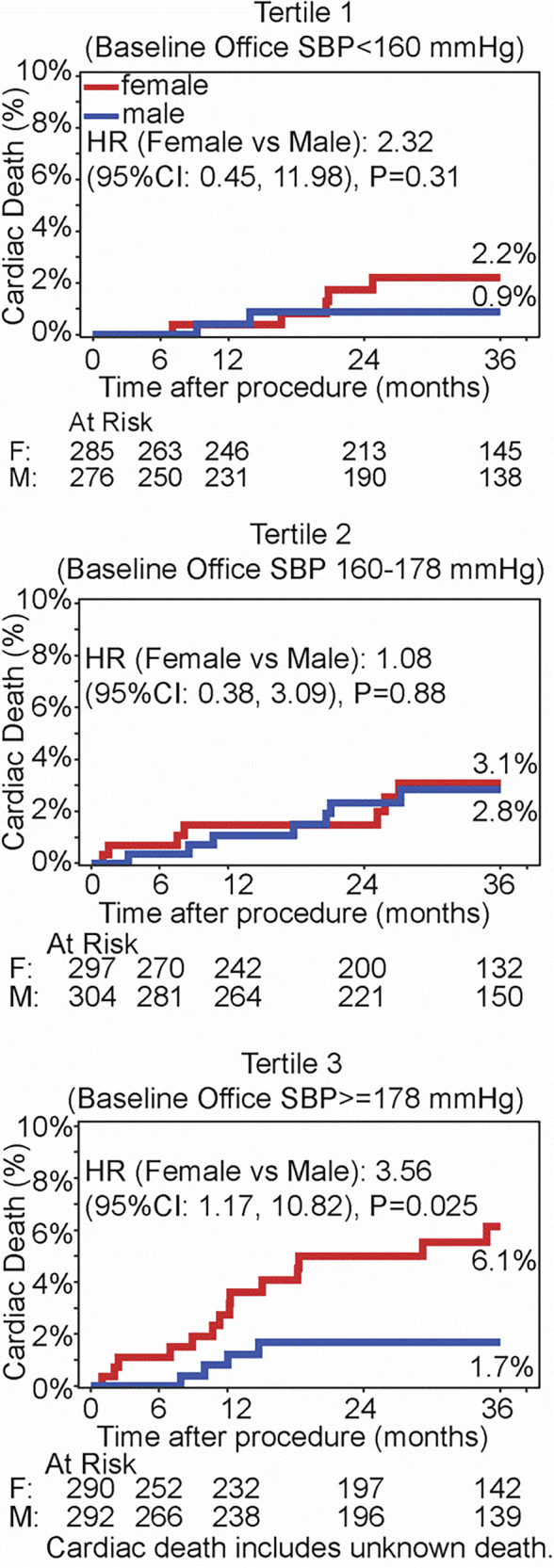
Cardiac death rates through 36 months in resistant hypertension patients by different baseline office systolic BP tertiles. Women and men were propensity matched. Kaplan Meier survival estimates were compared by sex in different baseline office systolic BP tertiles:  < 160 mmHg (top), 160 to 178 mmHg (middle), >=178 mmHg (bottom)

In the propensity-matched cohorts, there was no significant sex difference for outcome rates for all-cause death, stroke or myocardial infarction at 36 months (Fig. 3). However, there were more cardiac deaths in women than men at 36 months (HR 2.0; 95% CI 1.0 to 3.9; log rank *p* = 0.038). To further evaluate this discrepancy between sexes, we investigated clinical events by baseline blood pressure. Patients were stratified into tertiles based on their office SBP at baseline. Women within the upper tertile of baseline office SBP (>178 mmHg) had a significantly higher rate of cardiac death compared with men (6.1% vs 1.7%; HR 3.6 [95% CI: 1.2 to 10.8], *p* = 0.025; Fig. 4). No significant differences by sex were observed in lower BP tertiles.

To identify why women within the highest tertile of baseline office SBP were at greater risk for cardiac death than men, we investigated whether differences in treatment-related factors between groups may have influenced outcomes (Supplementary Table S6). For women in the upper tertile, there were statistically but not clinically significant differences in prescribed medication classes compared with men at baseline (5.0 ± 1.4 vs. 5.2 ± 1.4; *p* = 0.033) and at 36 months (4.7 ± 1.6 vs. 5.0 ± 1.6; *p* = 0.026). The proportion of prescribed antihypertensive medication classes was mostly balanced between groups, with the exception of vasodilators, which were prescribed more often to men than women. In this higher tertile of baseline SBP, men were more likely to be prescribed antidiabetic, antiarrhythmic, lipid-lowering treatments, and anticoagulants compared with women. Renal function decreased similarly in women and men through 36 months (female -6.5 ± 16.7 vs. -6.3 ± 18.9 mL/min/1.73m^2^), consistent with prior reports of a hypertensive population.

### Quality of life

In the propensity-matched cohort, there were significant sex differences in the quality-of-life (QoL) measures at baseline (Table 2). Women with rHTN had “some or severe problems” in all QoL parameters assessed, significantly more than men, including mobility, self-care, usual activities, pain or discomfort, anxiety, and depression. The overall EQ5D score for women was less than of men, as well as women’s self-reported “health state today”. Thirty-six months after RDN, the most significant improvement in QoL was observed in anxiety/depression, with women with rHTN showing a 12% improvement compared with baseline (p < 0.05).

**Table 2:** Self-assessed EQ5D questionnaire on quality of life at baseline in the propensity-matched cohort

% ± STD	Male (*n* = 872)	Female (*N* = 872)	*P* Value
EQ5D score	0.89 ± 0.18	0.83 ± 0.21	<0.0001
Your own health state today (%)	70.3 ± 17.5	63.6 ± 18.4	<0.0001
**Mobility**			
No problems	76.8	66.3	<0.0001
Some/severe problems	23.2	33.7	<0.0001
**Self-care**			
No problems	94.2	89.0	0.0003
Some/severe problems	5.8	11.0	0.0003
**Usual activities**			
No problems	77.2	62.5	<0.001
Some/severe problems	22.8	37.5	<0.001
**Pain/discomfort**			
No problems	58.8	41.4	<0.0001
Some/severe problems	41.2	58.6	<0.0001
**Anxiety/depression**			
No problems	72.7	52.5	<0.0001
Some/severe problems	27.3	47.5	<0.0001

## Discussion

Our analysis of this global registry of rHTN patients who received RDN provides unique insights into real-world management of severe hypertension based on sex. There were 42% women and 58% men enrolled from Western Europe, Eastern Europe, Southern Europe, the Middle East & Africa, the Americas, Asia, and the Pacific. Similar to most cardiovascular studies, women were older than men. We found other important differences in demographics at baseline. Women with rHTN had lower rates of smoking, sleep apnea, and heart disease, while BMI was comparable to men. In contrast, women had higher baseline office SBP, but not DBP, with higher pulse pressure and heart rate for women with rHTN. Increased pulse pressure and decreased DBP are indicators of arterial stiffness [21]. Arterial stiffness increases the risk for cardiovascular disease not only because it leads to myocardial stiffening [22] but also because it enhances the damaging effect of pulsatile pressure on the vessel wall. Confirmed in a recent report, finding women but not men, with elevated BP or hypertension in early midlife (42 years) is associated with increased arterial stiffness 27 years later [23]. The were small differences in eGFR that were statistically significant due to the large sample size, but lack clinical relevance.

A strength of this registry is that it also includes guidelines-recommended ambulatory blood pressure monitoring. At baseline, we found women with rHTN had higher 24-hr ambulatory SBP, lower 24-hr ambulatory DBP, and higher 24-hr heart rate, while non-dipping was more prevalent in men. Increased 24-hr heart rate for women and non-dipping BP profile for men is consistent with previous reports on ABPM [24]. Higher night-time BP for men is also consistent with the higher prevalence of obstructive sleep apnea in men (Table 1). Further sex differences in the regulation of muscle sympathetic nerve activity (MSNA) have also been reported [25]. For women, MSNA is linked to the level of blood pressure, whereas for men, it is linked to body mass index (BMI) and not BP. There is also a sharper age-related increase in MSNA in women than men, independent of menopausal status [26].

Our analysis from this global registry showed that the proportion of prescribed antihypertensive drug classes was similar between men and women with rHTN. As a real-world global registry, it also identified that only 83% of participants were taking diuretics as recommended by current guidelines for the treatment of rHTN. This once more highlights the known inertia (clinical or patient) previously reported for the treatment of hypertension. Women with rHTN were less likely to be prescribed a calcium channel blocker but more likely to receive spironolactone. These findings are in line with previous reports that women have more adverse drug events associated with calcium channel blockers [27], whereas men report more side effects to mineralocorticoid receptor antagonists. In women, in particular, calcium channel blockers were associated with leg edema [28]. Of note, previous studies report less adherence to antihypertensive medications for women with rHTN [12–14].

We used propensity-matched analysis to account for the significantly different baseline demographics between women and men was used to identify sex-specific differences in BP response to RDN and outcomes. Both women and men had significant BP reductions from baseline through to 36 months of comparable magnitude. Multivariate stepwise regression analysis identified that baseline SBP was the only significant and consistent independent factor (from both office and 24-h ambulatory BP) associated with SBP decreases in women with rHTN. By 36 months, the number of antihypertensive drug classes remained comparable by sex in patients with rHTN, with fewer women being prescribed calcium channel blockers, ACE inhibitors, and alpha-adrenergic blockers.

Sex differences in the self-assessed QoL questionnaire were identified at baseline in the propensity-matched cohort. Women indicated poorer self-care, mobility, anxiety, and depression compared with men. Depression has been shown to be an independent risk factor for cardiovascular disease in women [29]. Following 36 months after RDN, women with rHTN showed a significant 12% reduction in anxiety/depression compared to baseline. Women with rHTN were further evaluated by age, with a selection of <55 or >=55 years as the cutoff in line with increased arterial stiffness and pulse pressure increase at around the age of 55 years [30, 31]. The older group would also necessarily have a greater proportion of women who were postmenopausal.

Clinical outcomes were also evaluated after stratifying patients into tertiles based on their baseline office SBP. Interestingly, there was a significant disparity in the rate of cardiac death rate, which was significantly more prevalent in women within the upper tertile of baseline office systolic BP. This is consistent with a recent study, which showed increased CVD mortality risk for women compared with men for the same level of systolic BP [8]. Another study found that women, rather than men, had higher invasively measured central aortic systolic BP for the same cuff SBP [32]. It has been postulated that women with hypertension are more at risk than men for CVD mortality when co-morbid with autoimmune diseases, diabetes, obesity and more prone to iron deficiency, further putting them at higher CVD risk [33]. Moreover, women have smaller coronary arteries and aortic root dimensions compared with men, even after adjusting for body size discrepancies [34, 35]. Furthermore, women have higher arterial elastance and pulse pressure, stiffer and smaller aortic arches, and earlier wave reflection compared with men [36]. On top of these pronounced physiological differences that increase rHTN women’s CVD risk, postmenopausal women have higher autonomic nervous system activity compared with premenopausal women [37]. Each of these factors is not mutually exclusive and collectively contributes to the increased CVD risk observed in rHTN women.

### Strengths and Limitations

The strength of our analysis is that we determined outcomes based on longitudinal assessment, which is provided by registries, compared with time-limited clinical trials. Secondly, the propensity-matched analysis allowed us to minimize the differences in baseline demographics, providing important insights into the increased risk for CVD for women with rHTN. While our analysis identifies important sex-specific (biological) differences, further studies also need to consider gender (sociocultural) differences. Sex assigned at birth may not align with gender identity, with a growing transgender, gender-diverse, and nonbinary populations which have differing cardiovascular risk. There are also some limitations. As this was not a randomized study to compare outcomes in men vs. women with rHTN following RDN, outcome data are likely to be under-reported, with enrollment based on local referral patterns, preferences biased towards men. The GSR is an all-inclusive registry, with a broad range of patients of different nationalities with multiple comorbidities, reflecting the real world. In addition, the GSR has a significant proportion of patients who were female, which enabled this comprehensive sex-specific analysis. However, results are interpreted with caution as follow-up data were not available consistently in all patients. Further, the date of hypertension diagnosis is only available for a subset of the cohort enrolled (*N* = 1900). Most RDN patients have been treated for HTN for many years prior to the procedure, with a mean duration of 16.3 years.

There was no information on the reasons for lower recruitment of women with rHTN, whether it was physician inertia or patient preference [38]. However, our findings are supported by a recent report [39] that women were more likely to have delayed treatment of hypertension, which was associated with a higher risk of CV mortality. Secondary causes for hypertension or white coat hypertension were not excluded as part of the inclusion criteria. Further, women with rHTN enrolled in this registry may also have had a history of hypertensive disorders of pregnancy, but these data were not recorded. Also, not all patients had follow-up through 36 months with both office and 24-h ambulatory monitoring. Additionally, adherence to prescribed antihypertensive medications was not objectively assessed.

## Conclusion

Our analysis from the real-world global Symplicity registry has identified important sex differences in the phenotype of rHTN. Women with rHTN were older than men, potentially suggesting a delay in referral for a procedural intervention. They had lower QoL with a higher risk of cardiac death driven by higher baseline systolic BP. Following 36 months of receiving RDN, men and women with rHTN have significant BP reductions and significant improvement in anxiety and depression. Our findings support the need for prospective sex-specific analyses and for including more women in cardiovascular clinical trials, especially the understudied subpopulation of women with rHTN. The lower recruitment of women with resistant hypertension also highlights that we need to identify whether there is physician or patient inertia and encourage engagement. Our analysis highlights the need for specific early detection and a tailored approach to hypertension management for women.

## Supplementary information

Supplementary Materials

## Data Availability

The authors declare that all supporting data are available within the article and its Supplemental Material.
