# Supplementary material for: Sex stratified analysis of patients with resistant hypertension from the Global SYMPLICITY Registry of renal denervation
Source: Hypertens Res. 2025 Nov 20;49(3):904–15. doi: 10.1038/s41440-025-02446-y (PMC12960198; doi:10.1038/s41440-025-02446-y)

**Supplemental Materials**

**Sex Stratified Analysis of Patients with Resistant Hypertension from the Global Symplicity Registry of Renal Denervation**

Anastasia S. Mihailidou PhD^1,2^, Felix Mahfoud MD^3,4^, Markus Schlaich MD^5^, Roland Schmieder MD^6^, Krzysztof Narkiewicz PhD^7^, Luis Ruilope PhD^8^, Martin Fahy MS^9^, Michael Böhm MD^10^, Giuseppe Mancia PhD^11^, Laura Nickel MD^12^, Douglas A. Hettrick PhD^9^, Joachim Weil MD^12^

^1^Royal North Shore Hospital & Kolling Institute

^2^Macquarie University, Sydney, Australia
^3^Department of Cardiology, University Heart Center, University Hospital Basel, Switzerland

^4^Cardiovascular Research Institute Basel (CRIB), University Heart Center, University Hospital Basel, Switzerland

^5^Dobney Hypertension Centre, Medical School –Royal Perth Hospital Unit
^6^University Hospital Erlangen, Erlangen, Germany
^7^Medical University of Gdansk, Gdansk, Poland
^8^Hospital Universitario 12 de Octubre and CIBERCV and School of Doctoral Studies and Research, Universidad Europea de Madrid, Madrid, Spain
^9^Medtronic PLC, Santa Rosa, CA, USA

^10^Saarland University Hospital, Homburg/Saar, Germany
^11^University of Milano-Bicocca, Milan, Italy
^12^Sana Kliniken Lübeck GmbH, Lübeck, Germany

Short title: Phenotyping Resistant Hypertension in women

**Supplemental Table S1. Matched baseline propensity covariates**

|  | UNMATCHED | | | PROPENSITY 1:1 MATCHED | | |
| --- | --- | --- | --- | --- | --- | --- |
| Propensity Covariates | MALE | FEMALE | P-VALUE | MALE | FEMALE | P-VALUE |
| Office systolic blood pressure (mmHg) | 168.9 ± 19.4 (N=1440) | 174.2 ± 23.4 (N=1061) | <0.0001 | 170.9 ± 20.4 (N=872) | 171.3 ± 21.3 (N=872) | 0.70 |
| Number of antihypertensive meds | 5.1 ± 1.5 (N=1440) | 5.1 ± 1.5 (N=1061) | 0.87 | 5.1 ± 1.5 (N=872) | 5.1 ± 1.5 (N=872) | 0.58 |
| Age (yrs) | 59.2 ± 11.6 (N=1440) | 61.7 ± 12.7 (N=1061) | <0.0001 | 60.1 ± 11.7 (N=872) | 60.6 ± 12.8 (N=872) | 0.38 |
| Sleep apnea (%) | 25.0 (N=333/1331) | 12.5 (N=122/978) | <0.0001 | 14.7 (N=128/872) | 13.9 (N=121/872) | 0.68 |
| Cardiac disease (%) | 50.3 (N=719/1429) | 45.1 (N=474/1050) | 0.012 | 47.1 (N=411/872) | 46.6 (N=406/872) | 0.85 |
| Smoking history (%) | 41.3 (N=595/1439) | 19.1 (N=203/1061) | <0.0001 | 23.7 (N=207/872) | 21.6 (N=188/872) | 0.30 |

**Supplemental Table S2. Baseline and 36 months prescribed antihypertensive drug classes in propensity score matched cohorts of men and women**

| **Baseline antihypertensive drugs (%)** | **Males**  **(N=872)** | **Females**  **(N=872)** | **P-value** |
| --- | --- | --- | --- |
| Number of prescribed antihypertensive drug classes | 5.0 ± 1.4 | 4.9 ± 1.3 | 0.21 |
| ACE Inhibitors | 34 | 34 | 0.86 |
| Angiotensin Receptor blockers | 69 | 66 | 0.19 |
| Calcium Channel blockers | 82 | 79 | 0.11 |
| Diuretics | 83 | 83 | 0.99 |
| Mineralocorticoid Receptor antagonists | 29 | 31 | 0.37 |
| Spironolactone | 22 | 28 | 0.018 |
| Beta blockers | 81 | 80 | 0.45 |
| Alpha-adrenergic blocker | 40 | 37 | 0.30 |
| Centrally-acting sympatholytics | 40 | 37 | 0.36 |
| Direct renin inhibitors | 7 | 4 | 0.052 |
| Direct-acting vasodilators | 15 | 13 | 0.42 |
|  |  |  |  |
| **36 months antihypertensive drugs (%)** | **N=496** | **N=497** |  |
| Number of prescribed antihypertensive drug classes | 4.7 ± 1.6 | 4.7 ± 1.6 | 0.68 |
| ACE Inhibitors | 31 | 27 | 0.20 |
| Angiotensin Receptor blockers | 65 | 68 | 0.22 |
| Calcium Channel blockers | 82 | 76 | 0.026 |
| Diuretics | 77 | 80 | 0.27 |
| Mineralocorticoid Receptor antagonists | 33 | 33 | 0.86 |
| Spironolactone | 25 | 30 | 0.079 |
| Beta blockers | 77 | 74 | 0.31 |
| Alpha-adrenergic blocker | 36 | 32 | 0.17 |
| Centrally-acting sympatholytics | 32 | 35 | 021 |
| Direct renin inhibitors | 4 | 4 | 0.99 |
| Direct-acting vasodilators | 13 | 10 | 0.16 |

**Supplemental Table S3. Multivariate stepwise selection of significant baseline characteristics associated with BP changes at 36 months in women with rHTN**

| **Baseline covariates significantly associated with office systolic BP changes at 36 months (N=466)** | **Estimate** | **Standard Error** | **P value** |
| --- | --- | --- | --- |
| Intercept | 129.69 | 10.93 | <.0001 |
| Baseline Office SBP | -0.80 | 0.05 | <.0001 |
| Age | -0.19 | 0.10 | 0.044 |
| **Baseline covariates significantly associated with 24-h ambulatory systolic BP changes at 36 months (N=164)** | **Estimate** | **Standard Error** | **P value** |
| Intercept | 26.21 | 12.93 | 0.044 |
| Baseline 24Hr SBP | -0.21 | 0.08 | 0.006 |

Independent covariates selected using stepwise selection algorithm with entry/stay criteria of p≤0.10 from the following list of covariates: baseline systolic BP, number of antihypertensive drugs, body mass index, age, cardiac disease, diabetes, baseline heart rate, sleep apnea, atrial fibrillation, hypertension, combined hypertension, smoking history, baseline eGFR

**Supplemental Table S4. Sub-analysis of patients with refractory hypertension (baseline office SBP ≥140 mmHg and prescribed ≥5 antihypertensive medications including mineralocorticoid receptor antagonist).**

| Refractory hypertension | ALL | MALE | FEMALE | Difference | P-Value |
| --- | --- | --- | --- | --- | --- |
| **OFFICE SBP** |  |  |  |  |  |
| Baseline (mmHg) | 173.88 ± 22.99 (N=479) | 171.6 ± 21.7 (N=273) | 176.9 ± 24.3 (N=206) | -5.4 (-9.5, -1.2) | 0.0114 |
| 36 months (mmHg) | 151.94 ± 26.79 (N=215) | 149.9 ± 26.8 (N=117) | 154.3 ± 26.7 (N=98) | -1.6 (-8.5, 5.4) | 0.6596 |
| Change at 36 months (mmHg) | -19.45 ± 27.62 (N=215) | -18.5 ± 26.9 (N=117) | -20.6 ± 28.5 (N=98) | -1.6 (-8.5, 5.4) | 0.6596 |
| **OFFICE DBP** |  |  |  |  |  |
| Baseline (mmHg) | 94.46 ± 18.96 (N=479) | 94.9 ± 17.1 (N=273) | 93.9 ± 21.2 (N=206) | 1.0 (-2.4, 4.5) | 0.5633 |
| 36 months (mmHg) | 84.47 ± 18.67 (N=214) | 84.0 ± 18.1 (N=117) | 85.1 ± 19.4 (N=97) | -0.2 (-4.6, 4.1) | 0.9134 |
| Change at 36 months (mmHg) | -8.05 ± 18.82 (N=214) | -7.8 ± 19.9 (N=117) | -8.4 ± 17.6 (N=97) | -0.2 (-4.6, 4.1) | 0.9134 |

**Supplemental Tabel S5. Post hoc analysis of patients with diastolic resistant hypertension defined as baseline office SBP<140 mmHg and DBP≥ 90 mmHg.**

| Diastolic rHTN - SBP<140 AND DBP>=90 | ALL | MALE | FEMALE | Difference | P-Value |
| --- | --- | --- | --- | --- | --- |
| **OFFICE SBP** |  |  |  |  |  |
| Baseline (mmHg) | 133.5 ± 6.5 (N=39) | 132.6 ± 7.5 (N=27) | 135.4 ± 2.3 (N=12) | -2.8 (-7.3, 1.7) | 0.2173 |
| 36 months (mmHg) | 137.0 ± 19.4 (N=18) | 140.8 ± 18.6 (N=13) | 127.1 ± 19.9 (N=5) | 13.7 (-8.5, 35.9) | 0.2088 |
| Change at 36 months (mmHg) | 3.9 ± 20.8 (N=18) | 8.3 ± 20.5 (N=13) | -7.5 ± 18.9 (N=5) | 13.7 (-8.5, 35.9) | 0.2088 |
| **OFFICE DBP** |  |  |  |  |  |
| Baseline (mmHg) | 94.6 ± 3.6 (N=39) | 94.6 ± 3.9 (N=27) | 94.5 ± 2.8 (N=12) | 0.1 (-2.4, 2.7) | 0.9072 |
| 36 months (mmHg) | 89.0 ± 14.4 (N=18) | 91.6 ± 14.0 (N=13) | 82.3 ± 14.9 (N=5) | 8.8 (-7.1, 24.7) | 0.2578 |
| Change at 36 months (mmHg) | -5.4 ± 13.9 (N=18) | -2.9 ± 12.8 (N=13) | -11.7 ± 16.2 (N=5) | 8.8 (-7.1, 24.7) | 0.2578 |

**Supplemental Table S6. Prescribed medications among men and women in the highest office systolic blood pressure tertile (>178 mmHg).**

|  | Baseline | | | 36 months | | |
| --- | --- | --- | --- | --- | --- | --- |
| Category | Female (n=269) | Male (n=273) | P-value | Female (n=164) | Male (n=162) | P-value |
| # of antihypertensive medication classes | 5.0 ± 1.4 | 5.2 ± 1.4 | 0.033 | 4.7 ± 1.6 | 5.0± 1.6 | 0.026 |
| ACE Inhibitors | 37% (100/269) | 38% (103/273) | 0.89 | 37% (60/164) | 36% (58/162) | 0.88 |
| Angiotensin Receptor blockers | 64% (173/269) | 69% (188/273) | 0.26 | 61% (99/164) | 64% (103/162) | 0.55 |
| Calcium Channel blockers | 76% (204/269) | 77% (209/273) | 0.84 | 73% (119/164) | 80% (129/162) | 0.13 |
| Diuretics | 82% (221/269) | 83% (226/273) | 0.85 | 81% (132/164) | 77% (125/162) | 0.46 |
| Aldosterone antagonists | 31% (84/269) | 32% (86/273) | 0.95 | 31% (50/164) | 39% (63/162) | 0.11 |
| Spironalactone | 29% (77/269) | 25% (68/273) | 0.33 | 29% (48/164) | 32% (51/162) | 0.66 |
| Centrally-acting sympatholytics | 38% (102/269) | 45% (124/273) | 0.077 | 40% (66/164) | 40% (64/162) | 0.89 |
| Direct renin inhibitors | 5% (14/269) | 8% (22/273) | 0.18 | 4% (7/164) | 5% (8/162) | 0.77 |
| Beta blockers | 82% (221/269) | 86% (236/273) | 0.17 | 74% (121/164) | 82% (133/162) | 0.070 |
| Alpha-adrenergic blocker | 40% (107/269) | 44% (119/273) | 0.37 | 29% (48/164) | 36% (59/162) | 0.17 |
| Direct-acting vasodilators | 12% (32/269) | 18% (50/273) | 0.037 | 8% (13/164) | 15% (24/162) | 0.050 |
| Antiplatelet treatment | 30% (80/269) | 36% (97/273) | 0.15 | 32% (53/164) | 33 % (54/162) | 0.85 |
| Antidiabetic treatment | 26% (70/269) | 36% (99/273) | 0.010 | 28% (46/164) | 39 % (63/162) | 0.038 |
| Antiarrhythmic treatment | 2% (5/269) | 4% (10/273) | 0.20 | 2% (3/164) | 7% (11/162) | 0.027 |
| Lipid Lowering treatment | 44% (119/269) | 57% (156/273) | 0.0027 | 53% (87/164) | 62% (101/162) | 0.089 |
| Anti-Coagulants | 22% (60/269) | 34% (93/273) | 0.0024 | 24% (40/164) | 32% (51/162) | 0.15 |

P-values represent Pearson chi-squared or Mann-Whitney-Wilcoxon test.

**Supplemental Figure S1. Diastolic BP changes by sex through 36 months.**

1. Office diastolic BP B) 24-h ambulatory diastolic BP. Baseline office diastolic BP was 90 mmHg for women and 93 for men. Baseline 24-h ambulatory diastolic BP was 85 for women and 88 for men. All DBP changes from baseline are p<0.0001. The p values above comparing DBP changes by sex are ANCOVA adjusted for baseline diastolic BP. DBP=diastolic blood pressure.


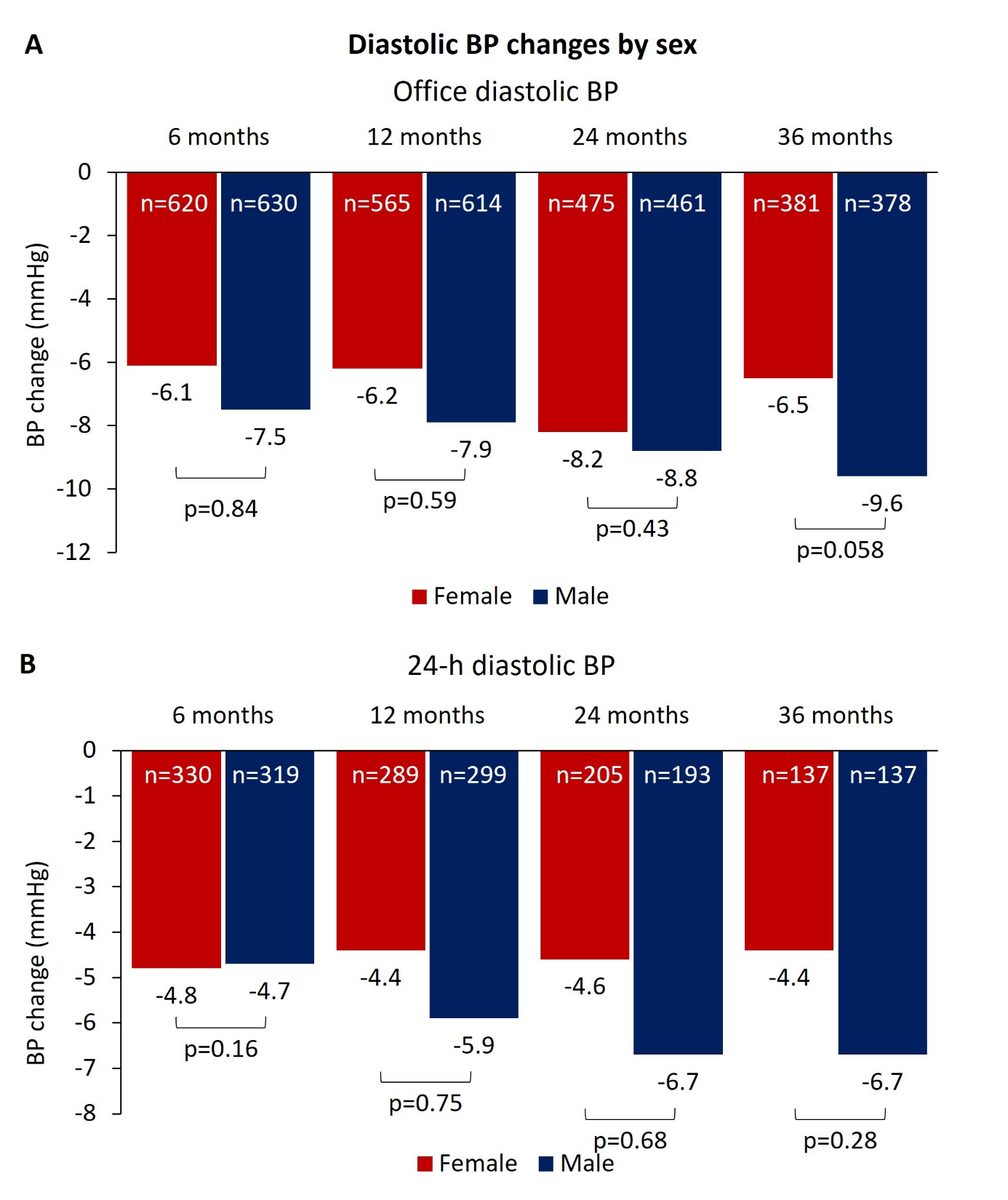


**Supplemental Figure S2. SBP changes through 36 months in women with resistant HTN by age groups.**

1. office systolic BP (<55yrs baseline: 174 mmHg; ≥55 yrs baseline: 169 mmHg). B) 24-h ambulatory systolic BP (<55yrs baseline: 159 mmHg; ≥55 yrs baseline: 153 mmHg). All SBP changes from baseline are p<0.0001. The p values above comparing SBP changes by sex are ANCOVA adjusted for baseline systolic BP. BP=blood pressure.


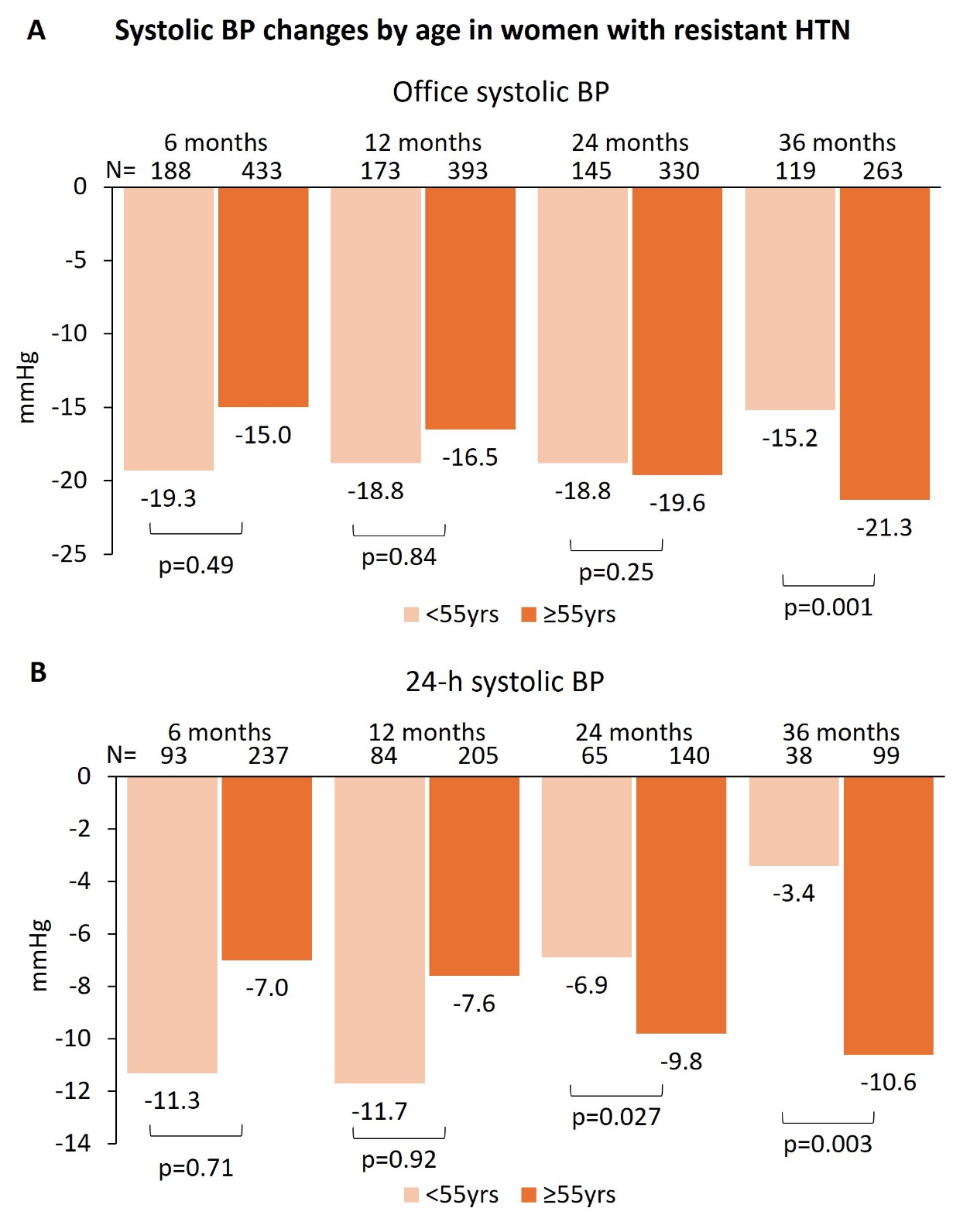

Supplement: Supplementary file 1 — Supplementary Materials [file 41440_2025_2446_MOESM1_ESM.docx]
